# Insights into the base-pairing preferences of 8-oxoguanosine on the ribosome

**DOI:** 10.1093/nar/gkz701

**Published:** 2019-08-10

**Authors:** Erica N Thomas, Carrie L Simms, Hannah E Keedy, Hani S Zaher

**Affiliations:** Department of Biology, Washington University in St. Louis, Campus Box 1137, One Brookings Drive, St. Louis, MO 63130, USA

## Abstract

Of the four bases, guanine is the most susceptible to oxidation, which results in the formation of 8-oxoguanine (8-oxoG). In protein-free DNA, 8-oxodG adopts the *syn* conformation more frequently than the *anti* one. In the *syn* conformation, 8-oxodG base pairs with dA. The equilibrium between the *anti* and *syn* conformations of the adduct are known to be altered by the enzyme recognizing 8-oxodG. We previously showed that 8-oxoG in mRNA severely disrupts tRNA selection, but the underlying mechanism for these effects was not addressed. Here, we use miscoding antibiotics and ribosome mutants to probe how 8-oxoG interacts with the tRNA anticodon in the decoding center. Addition of antibiotics and introduction of error-inducing mutations partially suppressed the effects of 8-oxoG. Under these conditions, rates and/or endpoints of peptide-bond formation for the cognate (8-oxoG•C) and near-cognate (8-oxoG•A) aminoacyl-tRNAs increased. In contrast, the antibiotics had little effect on other mismatches, suggesting that the lesion restricts the nucleotide from forming other interactions. Our findings suggest that 8-oxoG predominantly adopts the *syn* conformation in the A site. However, its ability to base pair with adenosine in this conformation is not sufficient to promote the necessary structural changes for tRNA selection to proceed.

## INTRODUCTION

Decoding of the genetic information is a remarkably accurate process that ensures the maintenance of faithful protein production. In all domains of life, the ribosome carries out this crucial task by utilizing multiple strategies to select for the aminoacyl-tRNA (aa-tRNA) that corresponds to mRNA in the A site (1,2). This process of tRNA selection is divided into two phases: initial phase and proofreading, which are separated by the irreversible step of GTP hydrolysis by EF-Tu (3). During the initial selection phase, aa-tRNA binds the A site of the ribosome in a ternary complex with EF-Tu and GTP. During this stage, near-cognate aa-tRNAs, which harbor a single mismatch, are discriminated against due to their inability to fully base pair with the A-site codon. This results in the accelerated dissociation of the ternary complex. After this initial codon-recognition step, EF-Tu undergoes a conformational change before GTP is hydrolyzed (4). This step of GTPase activation is significantly accelerated for cognate aa-tRNAs, thereby contributing to the overall accuracy of the tRNA selection process. After GTP hydrolysis, GDP-bound EF-Tu undergoes additional conformational changes before dissociating from the ribosome (5,6). During the subsequent proofreading stage, the selection process is partitioned into accommodation and rejection (7,8). Cognate aa-tRNAs rapidly accommodate to then participate in peptidyl transfer (PT), whereas near-cognate aa-tRNAs are more likely to be rejected (9–11). This multi-step process of tRNA selection results in an overall misincorporation rate of 10^−4^–10^−3^ per PT event (12–15)

X-ray crystallography and cryo-EM reconstitution studies of various ribosome complexes have provided some important molecular rationale for the process of tRNA selection, especially during the initial selection stage (16–18). The EF-Tu-bound aa-tRNA binds the A site in a bent state, referred to as the A/T state, where its anticodon can sample the codon (19). Once base pairing between the codon and the anticodon occurs, the conserved A1492, A1493 and G530 residues of the decoding center change conformation and interact with the minor groove of the codon–anticodon helix in a recently-identified stepwise manner (17). These interactions are only possible if strict Watson–Crick base pairing is maintained at the first two positions of the codon. Additional contacts are made by other ribosomal RNA (rRNA) residues as well as ribosomal protein S12 (20). These local rearrangements in the decoding center trigger a global change in the small ribosomal subunit (30S) (21). This so-called ‘domain closure’ moves the shoulder of the 30S as well as EF-Tu closer to the large subunit (50S). As a result, the GTPase domain of EF-Tu binds the sarcin-ricin loop (SRL), activating the factor for GTP hydrolysis through interactions with the catalytic histidine (22). It has been suggested that if the anticodon of the aa-tRNA is tightly bound in the decoding center following EF-Tu dissociation, accommodation ensues. On the other hand, if the tRNA is loosely bound then it is more likely to dissociate and be rejected (7,23). This ‘domain closure’ model for tRNA selection, however, has been called into question recently (24,25). Crystal structures of several ribosome complexes with near-cognate tRNAs revealed that mismatched base-pairs take on a Watson–Crick geometry and induce conformational changes nearly identical to those adopted in the presence of cognate tRNAs. In this ‘geometric selection’ model, discrimination against near-cognate aa-tRNAs is accomplished through energetic penalties associated with tautomerization of the bases to form Watson–Crick-like pairs with mismatched partners (24,25).

Several antibiotics are known to affect the overall selection process by altering the interactions between the tRNA–mRNA complex and the decoding center. The most studied and well-understood group is the aminoglycoside class of antibiotics. Nearly all bind in the decoding center and reduce the energetics of ‘domain closure’ by driving an ‘ON’-state of the decoding center nucleotides. For instance, paromomycin binds in a rRNA pocket close to A1492 and A1493 and induces them to adopt a structure similar to that assumed in the presence of cognate tRNAs. This, in turn, reduces the energetic cost associated with ‘domain closure’ of the 30S subunit and as a result, makes the process of tRNA selection more favorable in the presence of near-cognate aa-tRNAs (26,27). In comparison, streptomycin, which decreases GTPase activation for cognate aa-tRNA and increases it for near-cognate aa-tRNAs, does not induce ‘domain closure’. Instead, the antibiotic induces a lateral shift of helix 44 (h44), which contains A1492 and A1493; this rearrangement is distinct from that triggered by the addition of paromomycin. This lateral shift appears to be sufficient to stabilize near-cognate tRNAs, whereas the prevention of ‘domain closure’ destabilizes cognate tRNAs, which results in an overall increase in miscoding (28,29).

These largely structure-based models for tRNA selection, whereby local changes in the decoding center drive global rearrangements in the small subunit, are also supported by genetic studies. In particular, mutations in the 30S subunit that destabilize interactions that are important for the transition from the ‘open’ to ‘closed’ state result in a hyperaccurate phenotype. These mutations are typically found on the ribosomal protein S12, specifically at its interface with h27/h44 of the 16S rRNA near the decoding center (30,31). In contrast to the hyperaccurate mutants, error-prone (often referred to as *ribosomal ambiguity* (*ram*)) mutants reduce the energetics for transitioning to the ‘closed’ state of the 30S by disrupting interactions important for maintaining the ‘open’ state (32). Mutations of this class are associated with changes to the interfaces between ribosomal proteins S4 and S5 that are held together through electrostatic interactions in the ‘open’ state. Therefore, disruption of these interactions eases the transition to the ‘closed’ state, even in the presence of near-cognate tRNAs (33,34).

Under typical circumstances, the ribosome only encounters mRNA composed of the four canonical nucleobases. In contrast, the tRNA anticodon is often modified, and these modifications impact how the anticodon base pairs with the codon. Similarly, mRNA appears to be modified, albeit to a lesser extent than tRNAs. The most abundant of these mRNA modifications include N^6^-methyladenosine (m^6^A), 5-methylcytosine (m^5^C) and pseudouridine (Ψ) (35). Although these modifications do not change the Watson–Crick-base-pairing capabilities of the nucleotides, they affect the decoding process. For example, m^6^A reduces the overall rate of peptide-bond formation by almost an order of magnitude (36,37). In contrast, the introduction of Ψ to mRNA has little effect on the speed of decoding but reduces accuracy on stop codons *in vitro* (38,39). Regardless of their effect on decoding, the biological implications of these modifications are currently not fully understood, namely due to their low stoichiometries on mRNAs.

In contrast to these potentially intentional modifications, chemical damage to the mRNA nucleobase is largely detrimental to the decoding process. Most damage adducts occur as a result of reactivity between the mRNA and endogenous or exogenous agents (40,41). Some of the most common nucleotide-damaging agents include ultraviolet light, alkylating agents, and reactive oxygen species (ROS). In particular, ROS are produced endogenously as byproducts of metabolic reactions and increase under stress conditions (42). Of the many potential ROS adducts, 8-oxoguanosine (8-oxoG) is noteworthy due to its high abundance relative to other oxidized nucleotides and its association with neurodegenerative disease (43,44). Furthermore, 8-oxoG significantly reduces the rate of peptide-bond formation to a point that it stalls protein synthesis and is likely to activate the process of no-go decay (NGD). Indeed, our group has shown that the introduction of 8-oxoG to the mRNA, independent of its position within the codon, slows down PT by three to four orders of magnitude (45). While the overall kinetic consequences of 8-oxoG on tRNA selection were recognized, the mechanistic details through which 8-oxoG interferes with translation remained unknown. Specifically, we were interested in understanding how 8-oxoG disrupted interactions with the anticodon within the decoding center of the ribosome.

**Figure 1.**
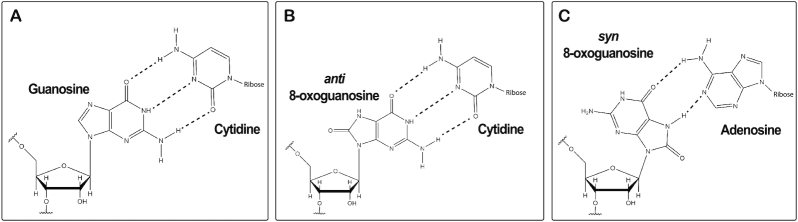
8-oxoG alters the base-pairing properties of the nucleotide. (**A**) Structure depicting the Watson–Crick base pair of unmodified guanosine and cytidine. (**B**) Structure of 8-oxoG in the *anti* conformation forming a Watson–Crick base pair with cytidine, and (**C**) in the *syn* conformation forming a Hoogsteen base pair with adenosine.

Previous data from studies of the oxidative damage of DNA show that 8-oxodG can alter the base pairing preferences of dG by changing the conformation of the nucleotide (46). When 8-oxodG adopts the typical *anti*-conformation, the oxygen at carbon 8 is in steric clash with the phosphate backbone (Figure 1). In order to relieve this steric clash, the base can rotate around its glycosidic bond to the *syn* conformation, where it reveals a new hydrogen-bonding interface which it uses to form a Hoogsteen base pair with dA (46). Different DNA polymerases read 8-oxodG as either a dG or dT at varying efficiencies, resulting in either accurate polymerization or a transversion. The efficiency of incorporating dCMP versus dAMP across 8-oxodG depends on the fidelity of the DNA polymerase. The steric constraints for base pairs in the active sites of high fidelity polymerases increase the frequency at which 8-oxodG base pairs with dA, as this base pair is nearly identical in terms of its geometry to a normal Watson–Crick base pair than 8-oxodG•C (47,48). While much is known about the base pairing preferences of 8-oxodG during replication, the preference for the *syn* vs *anti* conformation of the base on the ribosome is not understood at all.

In this study, we take advantage of a well-defined *in vitro* translation system to examine the mechanism through which 8-oxoG in mRNA interferes with translation (49). We find that 8-oxoG significantly impacts the initial phase of tRNA selection, suggesting that 8-oxoG is interfering with the ability of the A-site codon to form a proper interaction with its corresponding anticodon. To address how 8-oxoG is disrupting this interaction in the context of the A site, we explored its base-pairing preferences by relaxing tRNA-selection conditions and reacting it with its cognate tRNA and all possible near-cognate tRNAs. Under these relaxed tRNA selection conditions, we observed that 8-oxoG base pairs with either cytidine or adenosine independent of its location in either the first or second position of the codon. Our analysis also shows that 8-oxoG has a preference for base pairing with adenosine over cytidine under error-prone conditions, suggesting that it more frequently exists in the *syn* conformation than the *anti* one on the ribosome. Additionally, 8-oxoG disrupts the ability of the nucleotide to form base pairs with the remaining near-cognates (8-oxoG•U and 8-oxoG•G). Our results contribute to the mechanistic understanding of how 8-oxoG in mRNA disrupts translation.

## MATERIALS AND METHODS

### Materials

All reactions were performed in 1x polymix buffer (50), composed of 95 mM KCl, 5 mM NH_4_Cl, 5 mM Mg(OAc)_2_, 0.5 mM CaCl_2_, 8 mM putrescine, 1 mM spermidine, 10 mM K_2_HPO_4_ (pH 7.5), 1 mM DTT.

70S ribosomes were purified from MRE600 *Escherichia coli* via a double pelleting technique (34). Translation factors were overexpressed and purified from *E. coli* (49).

Modified mRNAs containing 8-oxoG were purchased from either IDT, Dharmacon, or The Midland Certified Reagent Company. Unmodified control mRNAs were transcribed from a dsDNA template using T7 RNA polymerase and purified via denaturing PAGE (51). The sequence for the first position 8-oxoG mRNA was as follows: CAGAGGAGGUAAAAAA AUG (8-oxo-rG)UU UUG UAC AAA. The sequence for the second position 8-oxoG-Arg mRNA was as follows: CAGAGGAGGUAAAAAA AUG C(8-oxo-rG)C UUGUACAAA. The sequence for the second position 8-oxo-Gly mRNA was as follows: CAGAGGAGGUAAAAAA AUG G(8-oxo-rG)C UUG UAC AAA.

### Charging of aminoacyl-tRNA

[^35^S]-fMet-tRNA^fMet^ was prepared as described (52). Pure tRNAs (tRNA^Val^, tRNA^Arg^, or tRNA^Met^ from ChemBlock) were aminoacylated by incubating them at 10 μM with the appropriate amino acid (0.4 mM), tRNA synthetase (∼5 μM) and ATP (2 mM) in charging buffer composed of 100 mM K-HEPES (pH 7.6), 20 mM MgCl_2_, 10 mM KCl, 1 mM DTT. After incubation at 37°C for 30 min, the aa-tRNAs were purified by phenol/chloroform extraction and ethanol precipitated. The aa-tRNAs were resuspended in in 20 mM KOAc (pH 5.2) and 1 mM DTT. Other tRNAs were aminoacylated by incubating total tRNA mix (Roche) at 150 μM in the presence of the corresponding amino acid and tRNA synthetase as above. The incubation and purification were conducted as that done for the pure tRNAs.

### Formation of ribosomal initiation complexes

Protocols were performed as described (53). Briefly, to generate initiation complexes (IC), the following components were incubated at 37°C for 30 min: 70S ribosomes (2μM), IF1, IF2, IF3, [^35^S]-fMet-tRNA^fMet^ (3 μM each), mRNA (6 μM) in 1× polymix buffer in the presence of 2 mM GTP. The complexes were then purified away from free tRNAs and initiation factors over a 500 μl sucrose cushion composed of 1.1 M sucrose, 20 mM Tris–HCl pH 7.5, 500 mM NH_4_Cl, 0.5 mM EDTA and 10 mM MgCl_2_. The mixture was spun at 287 000 $ \times$g at 4°C for 2 h, and the resulting pellet was resuspended in 1× polymix buffer and stored at –80°C. In order to determine the concentration of IC, the fractional radioactivity that pelleted was measured.

### GTP Hydrolysis assay

To assemble the ternary complexes, the following components were combined and incubated at 37°C for 15 min: 5 mCi/ml of [γ-^32^P]-GTP, 20 μM EF-Tu, and 5 μM of unlabeled GTP. An equal volume of 30 μM aa-tRNA was then added to the reaction and allowed to incubate again at 37°C for 15 min. In order to purify away unbound GTP and aa-tRNA from the assembled ternary complexes, samples were passed twice over P-30 spin columns (Biorad). The ternary complex was then diluted to 1 μM in polymix buffer (0.5 μM in the final reaction) and mixed with an equal volume of 2 μM IC (1 μM in the final reaction) at 20°C in a quench-flow instrument (RQF-3, KinTek Corporation). The reactions were quenched through the addition of 40% formic acid. The inorganic phosphate product was separated from unreacted GTP using Polyethylenimine (PEI) cellulose thin-layer chromatography (TLC) (Sigma) with 0.5 M potassium phosphate buffer pH 3.5 as a mobile phase. Fractional radioactivity corresponding to inorganic phosphate at each time-point was quantified using phosphorimaging and used to determine the observed rates of GTP hydrolysis.

### Kinetics of peptidyl transfer

EF-Tu (30 μM final) was initially incubated with GTP (2 mM final) in polymix buffer for 15 min at 37°C to exchange the bound GDP for GTP. To form the ternary complex, the mixture was incubated with aminoacyl-tRNAs (∼6 μM) for 15 min at 37°C. For reactions performed in the presence of antibiotics, streptomycin (100 μM final) or paromomycin (10 μg/ml final) were added to this mixture. The ternary complex mixture was then combined with an equivalent volume of IC at 37°C either by hand or using RQF-3 quench-flow instrument. The reaction was stopped at different time points using KOH to a final concentration of 500 mM. Dipeptide products were separated from free fMet using cellulose TLC plates that were electrophoresed in pyridine-acetate at pH 2.8 (54). The TLC plates were exposed to a phosphor screen overnight, and the screens were imaged using a Personal Molecular Imager (PMI) system. These images were quantified, and the fraction of dipeptide fMet at each time point was used to determine the rate of peptide bond formation using GraphPad Prism.

## RESULTS

### 8-OxoG interferes with the initial phase of tRNA selection

Previous work from our group showed that the presence of 8-oxoG within the A-site codon, regardless of its position, has a drastic effect on the speed of translation and slight effect on accuracy. The modification reduced the PT rate by almost three orders of magnitude for cognate aa-tRNA, and slightly increased it for the near-cognate tRNAs interacting through 8-oxoG•A base pairs with the codon (45). We hypothesized that the adduct inhibits base pairing, and as a result, is likely to inhibit early stages of tRNA selection, particularly the codon-recognition step. For technical reasons, we could not directly measure the kinetics of this step. Instead, in order to address the potential effect of the modification on the initial phase of tRNA selection, we opted to measure the rate of GTP hydrolysis as it reports on the overall selectivity of initial selection (11). To accomplish this, we utilized a pre-steady-state-kinetics strategy in combination with our reconstituted *in vitro* bacterial translation system. This system allows us to monitor individual and specific amino-acid incorporation. Briefly, ternary complexes were generated by incubating EF-Tu with a specific aa-tRNA in the presence of radio-labeled [γ-^32^P]-GTP. Purified ternary complexes were then incubated with initiation complexes programmed with intact mRNAs or 8-oxoG-containing ones, and rates of GTP hydrolysis were determined by stopping the reaction at various points.

**Figure 2.**
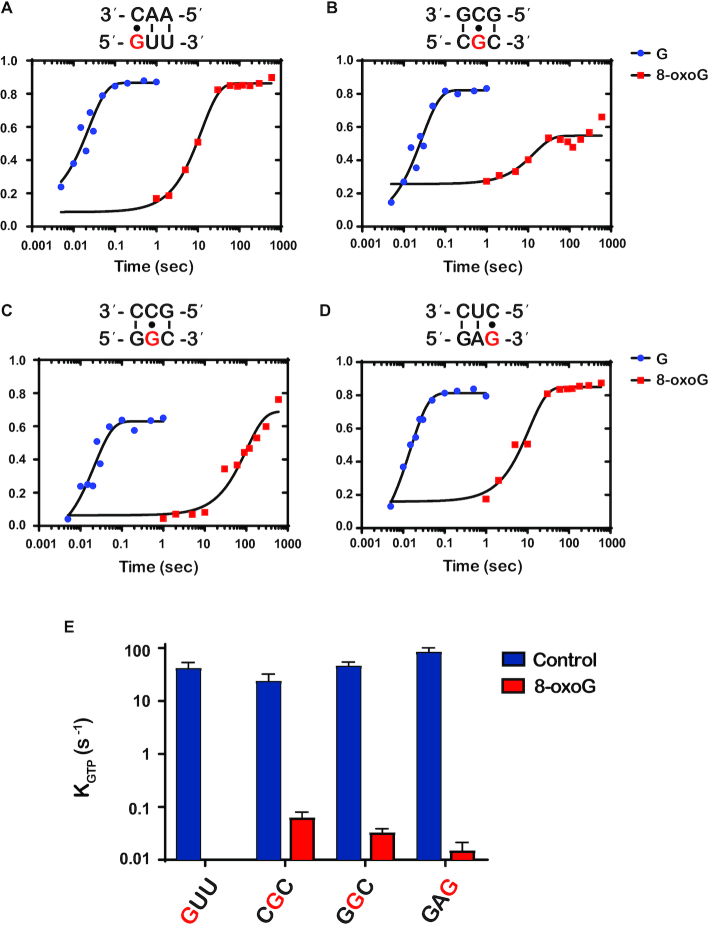
8-OxoG inhibits GTP hydrolysis by EF-Tu (**A**–**D**) Representative time courses of GTP hydrolysis reactions between the indicated initiation and ternary complex (codon is shown at the bottom, while the anticodon is shown at the top). For each codon, time courses were performed in the presence of G (blue) or 8-oxoG (red) and the position of 8-oxoG within the codon is indicated in red. E) Bar graph showing the observed rate of GTP hydrolysis (*k*_GTP_) for initiation complexes programmed with the indicated codon in the A site. 8-oxoG was introduced at the position depicted in red. Blue bars represent observed rates with unmodified complexes; red bars represent rates with 8-oxoG complexes. Plotted is the average of three independent experiments and the error bars represent the standard error of the mean.

In total, we analyzed eight different complexes harboring 8-oxoG at different positions of the A-site codon and their corresponding unmodified mRNAs. In particular, we measured the rates of GTP hydrolysis for the following complexes: ^8oxo^GUU, C^8oxo^GC, G^8oxo^GC and GA^8oxo^G, and the corresponding intact ones; these complexes code for Val, Arg, Gly and Glu, respectively. As predicted, we measured rates of GTP hydrolysis that were significantly lower for the oxidized mRNAs relative to the corresponding unmodified ones (> three orders of magnitude for three of the four complexes). More specifically, we measure rates of 42, 24, 47 and 86 s^−1^, respectively, whereas the same rates for the unmodified ones were <0.0001, 0.064, 0.033 and 0.015 s^−1^, respectively (Figure 2). Interestingly, this change in rates of GTP hydrolysis mirrors what we documented for PT, suggesting that most of the effects of the adduct on tRNA selection are due to alteration to the initial phase of the selection process.

### 8-OxoG impairs decoding in a manner similar to a mismatch with subtle but important distinctions

Thus far, our data has shown that the presence of 8-oxoG affects early stages of tRNA selection by potentially interfering with the codon–anticodon interaction. More specifically, we expect the modification to inhibit base pairing, resembling a mismatch. This would result in PT reactions with oxidized complexes behaving in a manner analogous to reactions involving near-cognate aa-tRNAs. To probe this prediction, we increased ribosomal promiscuity through the addition of aminoglycoside antibiotics to our *in vitro* PT reactions. This results in relaxed tRNA-selection parameters, which increases the incorporation of near-cognate aa-tRNAs; and based on our model, aa-tRNA reactivities should also increase with the oxidized complexes in the presence of these antibiotics.

**Figure 3.**
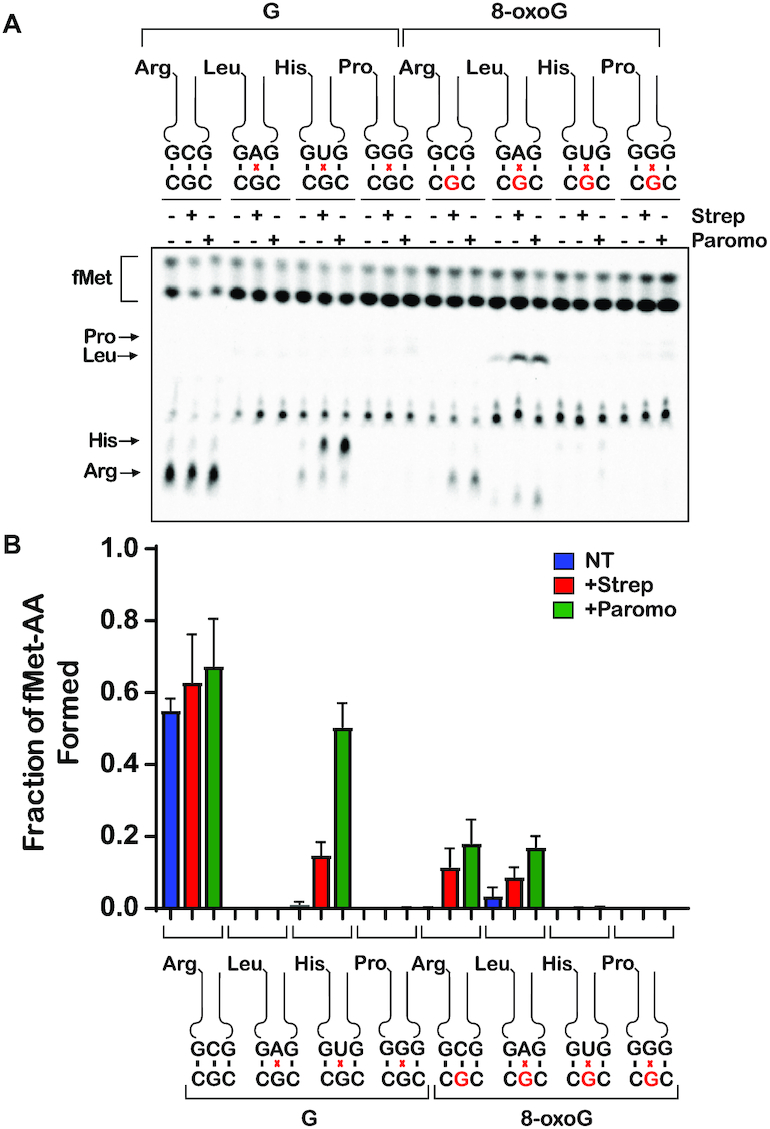
8-oxoG in the second position of the codon changes the base pairing properties of guanosine on the ribosome (**A**) Phosphorimager scan of electrophoretic TLCs used to follow dipeptide-formation reactions (5-second incubation time) in the presence of the indicated initiation and ternary complexes in the absence and presence of the indicated antibiotics. (**B**) Quantification of the dipeptide yield as performed in (A). Plotted is the average of three independent experiments and the error bars represent the standard deviations around the means.

We reacted the intact (CGC) complex and its oxidized counterpart (C^8oxo^GC) with its cognate Arg-tRNA^Arg^ ternary complex and every possible second-position-near-cognate ternary complex in the absence and presence of streptomycin or paromomycin. As expected, after 5 seconds of incubation, significant dipeptide formation was observed only in the presence of the cognate ternary complex for the intact CGC complex (Figure 3 and Supplementary Figure S1). Additionally, as we had reported earlier, the presence of 8-oxoG severely inhibited the formation of the cognate fMet-Arg dipeptide while increasing the incorporation of Leu-tRNA^Leu^, for which the second position A of the anticodon base pairs with the 8-oxoG. The addition of antibiotics had no effect on the cognate reaction in the presence of intact mRNA. However, and as anticipated, the antibiotic significantly increased the formation of only the near-cognate fMet–His dipeptide. This is rationalized by the fact that tRNA^His^ harbors U at the second position of its anticodon, which allows it to form a less deleterious wobble-base pair with the G of the mRNA’s codon (55). Consistent with our model that 8-oxoG changes the decoding process in a manner resembling that of a near-cognate, the addition of antibiotics to the C^8oxo^GC complex increased the formation of fMet-Arg dipeptide and that of fMet-Leu. Interestingly, the antibiotics appear to have little to no effect on the reactivity of the C^8oxo^GC complex with His-tRNA^His^. Together, these observations suggest that the effects of 8-oxoG on translation can be suppressed by antibiotics, but in a manner slightly distinct from a mismatch.

**Figure 4.**
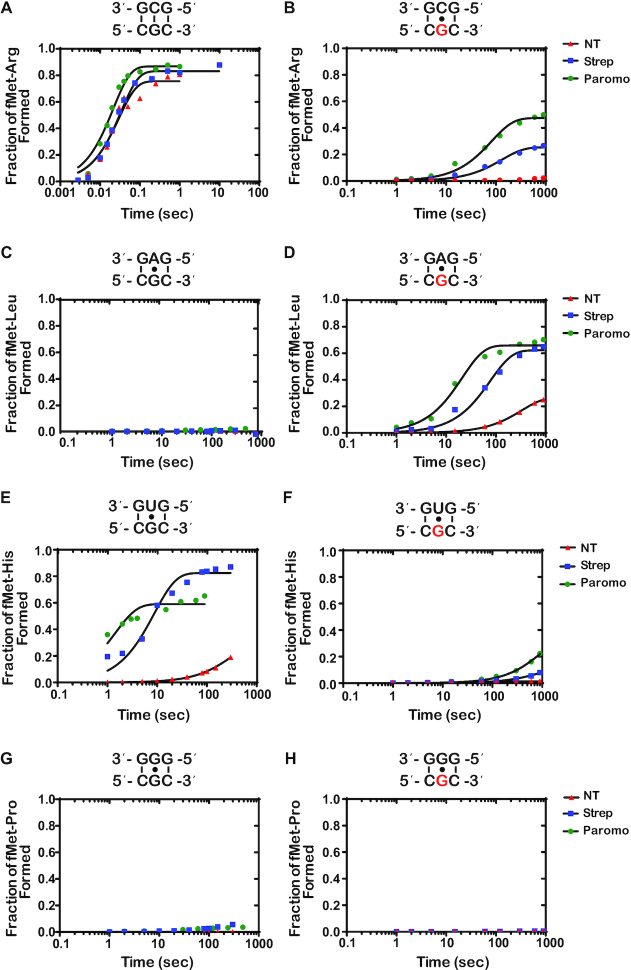
The addition of antibiotics increases the *k*_pep_ and Fp for cognate and a subset of near-cognate tRNAs in the presence of 8-oxoG at the second position of the codon (**A**–**H**) Representative time courses of peptide-bond-formation reactions between the indicated initiation and ternary complexes in the absence and presence of the indicated antibiotics. The time courses shown on the right panel were carried out with the unmodified complex, whereas ones shown on the left panel were carried out with the modified complex (8-oxoG drawn in red). Time courses were conducted at least in duplicates. We note that the observed rates varied from experiment to experiment due to differences in different preparation of aa-tRNA in the tRNA mix; the fold difference as a result of antibiotic addition, however, was reproducible.

To add more quantitative support for these differential reactivity profiles, we conducted full time courses of the PT reactions in the absence and presence of streptomycin or paromomycin (Figure 4). Performing full time courses allowed for us to measure the observed rate of peptide-bond formation (*k*_pep_) which reports on the combined rates of aa-tRNA accommodation (*k*_5_) and rejection (*k*_7_), as well as the end point of each reaction (Fp), which reports on the effectiveness of proofreading (*k*_5_ relative to *k*_pep_) (9). In agreement with our end-point analysis, the antibiotics had little effect on the endpoints and the rate of the reactions between the native initiation complex and the cognate aa-tRNA (Figure 4A). This is in slight disagreement with previous reports showing that streptomycin reduces the rate of peptide bond formation for cognate reactions by approximately twofold (28,56), whereas in our assays, streptomycin only slightly decreased these rates. We note that these earlier experiments utilized different buffer systems, for which the observed rate of peptide-bond formation in the presence of streptomycin is limited by GTP hydrolysis.

Interestingly, the addition of the antibiotics to the same reaction with the 8-oxoG-containing complex caused the observed rate of PT to increase twofold – we measured average rates of 0.0097, 0.022 and 0.049 s^−1^in the absence of antibiotic and in the presence of streptomycin or paromomycin, respectively (Figure 4B). Additionally, the endpoint of the reactions increased by approximately an order of magnitude in the presence of the antibiotics, with measured Fp values of 0.027, 0.37 and 0.52 for no antibiotic, streptomycin, and paromomycin, respectively. Next, we performed reactions in the presence of the near-cognate aa-tRNAs. We started with the G•A mismatch reaction involving the Leu-tRNA^Leu^ ternary complex. As our reactivity-survey assay indicated, the addition of antibiotics did not increase the rate or endpoint of PT with the intact complex but caused both to significantly increase for the oxidized complexes (Figure 4C and D). For the G•G and 8-oxoG•G mismatches involving Pro-tRNA^Pro^ ternary complex, the addition of streptomycin had a barely detectable effect on the PT rate (Figure 4G and H). In contrast, the antibiotics increased the observed PT rate for the His-tRNA^His^, which forms a wobble G•U mismatch with the mRNA, by more than an order of magnitude. Additionally, the Fp value for the same reaction increased by more than twofold as a result of antibiotic addition (Figure 4E). In contrast to the unmodified complex, His-tRNA^His^ failed to react with the 8-oxoG complex (Figure 4F). These observations suggest that the modification does not allow the mRNA to form a wobble base pair with U. Altogether, our findings suggest that oxidation of G changes the base-pairing preference for the modified nucleotides on the ribosome, likely due to its chemical nature as well as the geometry of the decoding center.

To provide further support for our model that antibiotics can suppress the effect of 8-oxoG on decoding, we tested another set of complexes that displayed a different codon in the A site. In particular, we programmed ribosomes with the oxidized G^8oxo^GC codon and tested their reactivity with the cognate Gly-tRNA^Gly^ and near-cognate Val-tRNA^Val^ ternary complexes. Similar to what we observed for the G^8oxo^GC complex, both streptomycin and paromomycin increased the rate of peptide-bond formation significantly (Supplementary Figure S2). These observations suggest that aminoglycosides suppress the effect of the modification independent of the codon identity.

### The base-pairing preferences of 8-oxoG at the first position of the codon are slightly different from those observed at the second position

**Figure 5.**
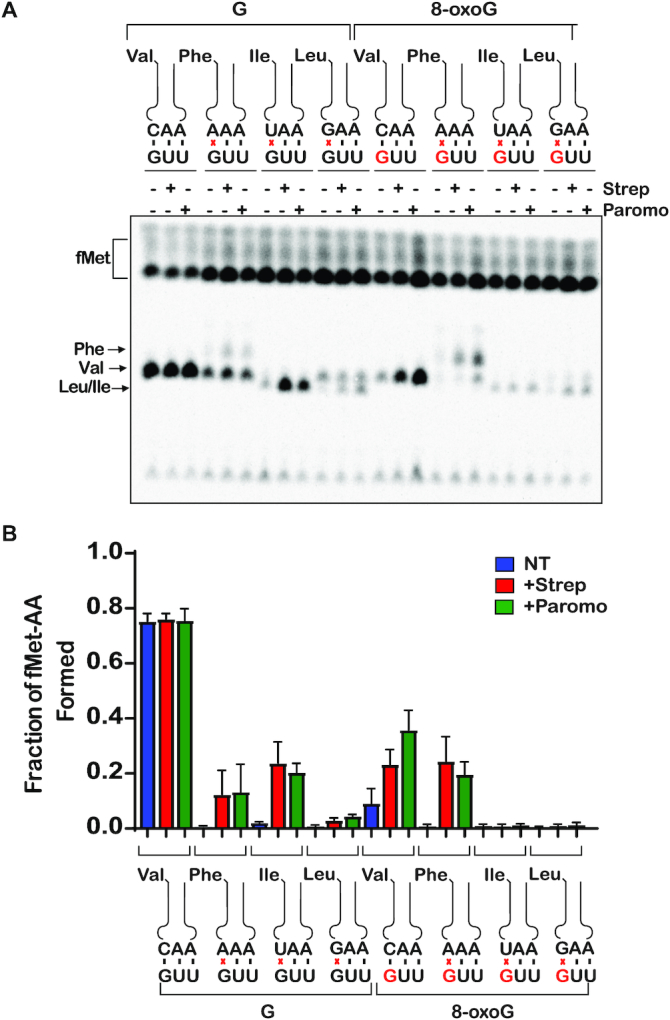
Antibiotics suppress the effects of 8-oxoG in the first position of the codon (**A**) Phosphorimager scan of electrophoretic TLCs used to follow dipeptide-formation reactions (5-second incubation time) in the presence of the indicated initiation and ternary complexes in the absence and presence of the indicated antibiotics. (**B**) Quantification of the dipeptide yield as performed in (A). Plotted is the average of three independent experiments and the error bars represent the standard deviations around the means.

Our data thus far shows that when 8-oxoG is in the second position of a codon, it changes the base-pairing preferences of G on the ribosome. In order to investigate if the base-pairing preferences of 8-oxoG that we observed were specific to the second position, we performed the same peptidyl-transfer experiments with a codon containing 8-oxoG at the first position. We reacted the complex containing intact codon (GUU) and the complex containing the 8-oxoG codon (^8oxo^GUU) with the cognate Val-tRNA^Val^, as well as all possible first-position-near-cognate aa-tRNAs in the presence of paromomycin or streptomycin. Once again, after 5 seconds of incubation with no antibiotic, significant amount of dipeptide was formed exclusively in the presence of the cognate ternary complex for the GUU codon, and the presence of 8-oxoG substantially decreased the formation of the cognate dipeptide (Figure 5). However, at this position and after 5 seconds of incubation, 8-oxoG did not result in any observable increase in the reactivity of the initiation complex with Phe-tRNA^Phe^, which has an A at the third position of the anticodon (Figure 5). This is contrary to what we observed in the second position, where dipeptide is formed in the presence of the 8-oxoG•A base pair without the addition of antibiotics (Figure 3). We note that our source of the tRNA mix often contained low levels of charged tRNAs, even after extensive attempts at deacylation. Therefore, we observed some residual reactivity with the cognate Val-tRNA^Val^ in reactions containing the near-cognate tRNAs such as Phe-tRNA^Phe^, but that did not affect our quantification since the two peptides migrate differently on our TLCs, allowing us to distinguish them.

Upon addition of antibiotics, we observed significant increases in dipeptide formation for the intact codon with two of the three near-cognate aa-tRNAs, namely Phe-tRNA^Phe^ and Ile-tRNA^Ile^, for which the third position of the anticodon is an A and a U, respectively. This differs from what we observed for the second-position mismatches, for which the addition of antibiotics increased the dipeptide formation only for the near-cognate tRNA with the G•U base pair. When we add the antibiotics to the reactions containing the 8-oxoG codon, we observe an increase in the incorporation of Val-tRNA^Val^ and Phe-tRNA^Phe^, for which the first position of the anticodon is a C and A, respectively. This is similar to what we observe for 8-oxoG in the second position of the codon. In both the first and second position of the codon, our data shows that 8-oxoG base pairs with adenosine as well as cytidine when tRNA selection is relaxed, suggesting that it is able to adopt both the *syn* or *anti* conformation on the ribosome.

**Figure 6.**
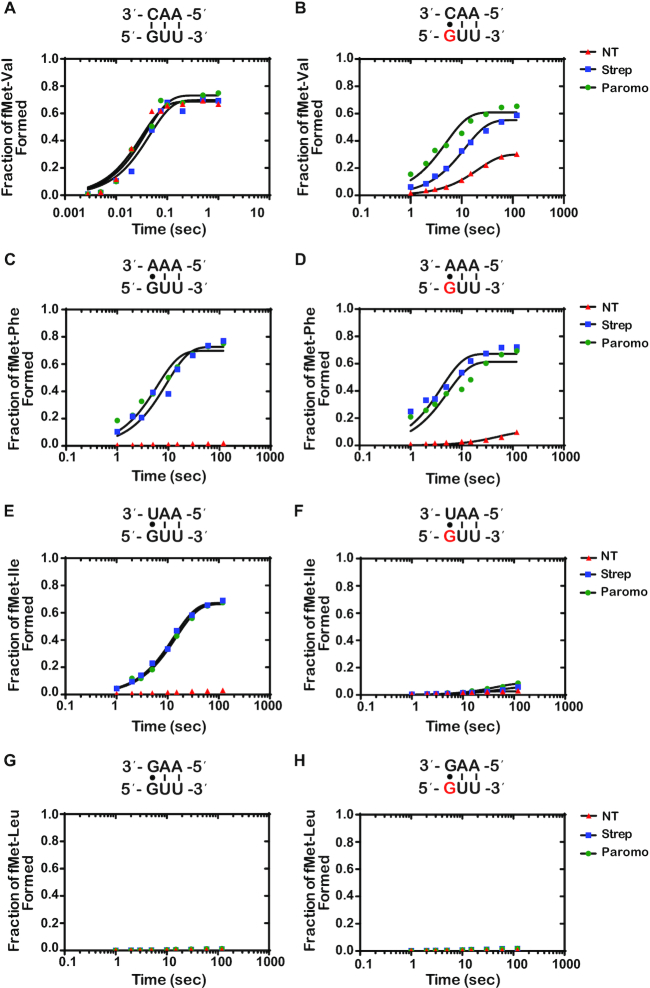
Antibiotics drastically increase *k*_pep_ and Fp for reactions between the ^8oxo^GUU complex with Phe-tRNA^Phe^ (8-oxoG•A base pair), and only slightly for reactions with Val-tRNA^Val^ (8-oxoG•C) (**A–H**) Representative time courses of peptide-bond-formation reactions between the indicated initiation and ternary complexes (codon is shown at the bottom, while the anticodon is shown at the top) in the absence and presence of the indicated antibiotics. The time courses shown on the right panel were carried out with the unmodified complex, whereas ones shown on the left panel were carried out with the modified complex (8-oxoG drawn in red). Time courses were conducted at least in duplicates; the fold difference as a result of antibiotic addition was reproducible.

Again, to provide additional quantitative support for our reactivity profiles, we performed full time courses of the PT reactions in the absence and presence of the aminoglycoside antibiotics (Figure 6 and Supplementary Figure S3). As expected, the addition of the antibiotics had no significant effect on the reaction of the intact GUU codon with the cognate Val-tRNA^Val^ (Figure 6A). We measured rates of 31, 21 and 25 s^−1^ and Fp values of 0.69, 0.72 and 0.73 for the no treatment, streptomycin, and paromomycin conditions, respectively. For the reactions between near-cognate Phe-tRNA^Phe^ and Ile-tRNA^Ile^ with the intact complex (G•A and G•U mismatches, respectively), the addition of antibiotics was found to result in an increase in the endpoint, but not the rate (Figure 6C and E). This is in direct contrast to what we observed for mismatches at the second position, for which the addition of the antibiotics substantially increased the rate and endpoint of PT for the G•U base pair only and no other mismatches (Figure 4E and C), consistent with the observations that decoding at the second position appears to be more stringent relative to that at the first one (36).

For the 8-oxoG-containing codon, we measured a rate and endpoint with Val-tRNA^Val^ (8-oxoG•C base pair) of 0.044 s^−1^ and 0.308, respectively. Both of these values are much higher than those measured for Phe-tRNA^Phe^ (8-oxoG•A mismatch); *k*_pep_ of 0.018 s^−1^ and Fp of 0.11 (Figure 6B and D). This differs from what we observed with 8-oxoG in the second position, where the rate and endpoint were higher for the reaction involving an 8-oxoG•A interaction relative to the 8-oxoG•C (Figure 4B and D). These observations could be explained by at least two scenarios: 1) the frequency of rotation of 8-oxoG around its glycosidic bond might be different depending on its position within the codon; 2) the rate of dissociation of Phe-tRNA^Phe^ from the 8-oxoGUU complex is slow, when 8-oxoG is in the *syn* conformation, allowing the tRNA to sample the *anti* conformation to form a Watson–Crick base pair and proceed with tRNA selection. Interestingly, the 8-oxoG•A Phe-tRNA^Phe^ reaction was found to benefit much more from the addition of antibiotics relative to the 8-oxoG•C Val-tRNA^Val^ reaction. For the Val-tRNA^Val^ reaction (8-oxoG•C base pair), the observed rate and endpoint increased by a mere twofold to fourfold in the presence of streptomycin and paromomycin (Figure 4B). In contrast, in the presence of Phe-tRNA^Phe^ (8-oxoG•A base pair), the observed rate increased by more than an order of magnitude and the endpoint increased by approximately sixfold (Figure 4D). These observations are consistent with the second scenario, whereby 8-oxoG prefers the *syn* conformation in the decoding center, but the addition of antibiotics stabilizes the tRNA long enough to allow it to sample the *anti* conformation. If the tRNA harbors a C at that position, the selection process can proceed.

### Error-prone and hyperaccurate ribosomes suppress and exaggerate the effects of 8-oxoG on decoding, respectively

**Figure 7.**
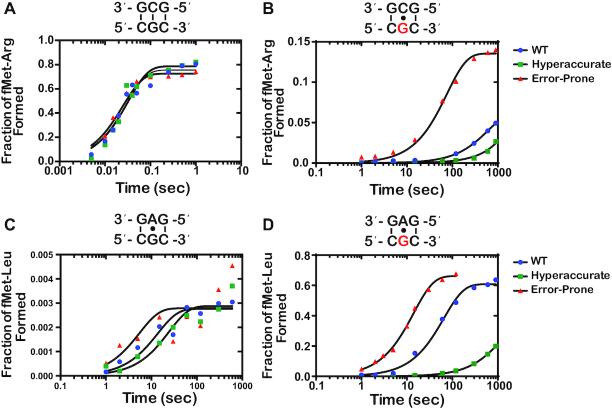
Hyperaccurate and error-prone ribosomes suppress and amplify the effects of 8-oxoG, respectively (**A–D**) Representative time courses of peptide-bond formation between the indicated initiation and ternary complexes with the depicted ribosome mutant. The time courses shown on the right panel were carried out with the unmodified complex, whereas ones shown on the left panel were carried out with the modified complex (8-oxoG drawn in red). Time courses were conducted at least in duplicates for constructs containing 8-oxoG; the fold difference was reproducible.

To provide further support for our model that altering tRNA selection parameters changes the effect of 8-oxoG on decoding independent of drug addition, we utilized error-prone as well as hyperaccurate ribosome mutants and assessed their effect on PT in the presence of 8-oxoG (Figure 7). We chose the well-studied *rpsD12* and *rpsL141* mutants as representatives for the error-prone and hyperaccurate types, respectively (57). As expected, the mutations had no effect on the observed PT rate or endpoints for the intact complex in the presence of the cognate aa-tRNA (Figure 7A). In contrast, and in agreement with our model, in the presence of 8-oxoG the error-prone mutation increased the observed rate of formation of fMet-Arg dipeptide by sevenfold, whereas the hyperaccurate decreased it by approximately fourfold (Figure 7B). Similarly, and consistent with their effect on decoding, the error-prone mutation slightly increased the observed PT rate for the near-cognate (G•A base pair), whereas the hyperaccurate one slightly decreased the rate (Figure 7C). In the presence of 8-oxoG, the hyperaccurate mutation suppressed the modification-induced misincorporation of Leu-tRNA^Leu^ (8-oxoG•A base pair), for which we observe an almost ninefold decrease in the observed PT rate, while the error-prone mutation increased the misincorporation by fivefold (Figure 7D). Interestingly, for both the no antibiotic and antibiotic treatments, the endpoints for PT reactions involving the 8-oxoG•A interactions were at least twofold relative to those measured for ones involving the 8-oxoG•C base pairs (Figure 7B and D). Collectively our data utilizing drug- as well as mutation-induced alteration of the tRNA-selection process support our model that 8-oxoG can base pair in either the *syn* or *anti* conformation in the context of the A site, with a preference for the *syn* conformation. Additionally, our data shows that 8-oxoG disrupts the ability of guanosine to mispair with uridine, suggesting that the lesion modifies the conformation in which guanosine can miscode.

## DISCUSSION

Recent reports from a number of groups have shown that modification of the mRNA occurs at levels that could potentially affect its function (35). Emerging from these studies are the observations that ribosomal function as well as the decoding process could be significantly altered as a consequence of these modifications. For some adducts, such as m^6^A which are deliberately modified by cellular enzymes, the modifications appear to play roles in regulating gene expression (58). In contrast, for most unintended adducts, like those that result from chemical damage, the modifications are a burden to the translation machinery and pose challenges to the speed and accuracy of the ribosome. We previously chose to study the effects of the oxidized base 8-oxoG due to its high prevalence, especially under certain conditions, as well as its unique chemical properties (43–46). Introducing the adduct to the mRNA, regardless of its position within the A-site codon, slowed down PT significantly. Previous studies regarding the impact of 8-oxoG on DNA replication show that the modification can increase C to A transversions by preferentially mispairing with A (59). Interestingly, 8-oxoG was found to only slightly increase misincorporation of near-cognate aa-tRNAs during translation. These findings suggested that 8-oxoG interferes with tRNA selection. Here, we expanded on these studies by characterizing the mechanism by which 8-oxoG affects the decoding process. A priori, we hypothesized that base-pairing interaction with the modified nucleotide resembles a mismatch. As a result, 8-oxoG fails to trigger the required conformational changes in the decoding center to proceed through the tRNA selection process. Consistent with this proposal, we find the modification to severely inhibit GTP hydrolysis by EF-Tu, suggesting that it affects the initial phase of the selection process (Figure 2). Furthermore, the introduction of miscoding antibiotics or ribosomes with error-prone mutations was found to partially rescue the effect of the modification, as would be expected if 8-oxoG•C and 8-oxoG•A base pairs were to resemble mismatches. Indeed, when we add the antibiotics to the reactions of intact codons and near-cognate aa-tRNAs, we see similar increases in *k*_pep_ and/or Fp.

While the 8-oxoG•C and 8-oxoG•A base pairs resemble mismatches in both the first and second position of the codon, we observe that 8-oxoG has distinct base-pairing preferences based on its position within the codon. In the absence of antibiotics, 8-oxoG in the second position prefers to base pair with A, while 8-oxoG in the first position prefers to base pair with C (Figures 4 and 6). Structural studies of the A site show that the interactions between the second position codon and its corresponding anticodon are monitored by the universally conserved A-minor interactions of A1492, as well as G530 of the 16S rRNA and S50 of the ribosomal protein S12. Meanwhile, the interactions between the first position codon and its corresponding anticodon are only monitored by the A-minor interactions of A1493. Monitoring at both positions works to ensure that only Watson–Crick base pairs are recognized as acceptable interactions (17,20,21). We speculate that the bulky conformation of the *anti*-8-oxoG•C base pair is not recognized as an acceptable interaction in the highly-monitored second position, thus explaining why we observe a preference for the *syn*-8-oxoG•A. Alternatively, the *anti* conformation of the modified base might be very short lived that during codon recognition tRNAs harboring a C at the second position dissociate before they can sample it. In the less stringently monitored first position, we observed a preference for the *anti*-8-oxoG•C base pair in the absence of antibiotics, which could be explained by decreased dissociation rates for near-cognate tRNAs at this position (60).

Upon addition of antibiotics, the average *k*_pep_ and Fp for the 8-oxoG•A in the first position exceeded that of 8-oxoG•C, more closely resembling the trends we observe in the second position of the codon (Figures 4 and 6). Additionally, we observe higher *k*_pep_ and/or Fp values for 8-oxoG•A than 8-oxoG•C in the presence of error-prone ribosomes. Previous studies have shown that 8-oxoG prefers to exist in the *syn* conformation because of steric repulsion between the 8-oxo and the phosphate backbone (46). This is consistent with our model that 8-oxoG primarily exists in the *syn* conformation on the ribosome, and under normal conditions, is recognized as a mismatch when it base pairs with A and is rejected during codon recognition. When error-prone conditions, which suppress the effect of mismatches, are introduced in this case, the 8-oxoG(syn)•A base pair can move through codon recognition and into proofreading, and its Fp values are almost restored to those observed in the presence of a G•U base pair (Figure 7). We speculate that 8-oxoG does not exist in the *anti* conformation as frequently, but upon addition of antibiotics, the codon–anticodon interaction is stabilized long enough to allow for the 8-oxoG to change from the *syn* to the *anti* conformation and proceed with codon recognition.

The hypothesis that 8-oxoG primarily exists in the *syn* conformation on the ribosome is also supported by its ability to disrupt base pairing with U. We observe significant reductions in *k*_pep_ and/or Fp values in the presence of an 8-oxoG•U base pair in error-prone conditions compared to G•U, regardless of its position within the codon (Figures 4E, F, and 6E, F). This was a surprising observation because the G•U wobble conformation should not be disrupted by the introduction of the oxygen at carbon 8; therefore, we expected to see an increase in 8-oxoG•U mispairing in the presence of antibiotics. In order to form a wobble base pair with U, 8-oxoG needs to be in the *anti* conformation (55). We speculate that the inability of the antibiotics to increase miscoding in the presence of 8-oxoG•U is due to 8-oxoG primarily existing in the *syn* conformation on the ribosome. Additionally, we cannot exclude the possibility that the 8-oxoG•U wobble base pair is structurally unfavorable in the A site; however, further studies would need to be performed to test this hypothesis.

Interestingly, circular dichroism (CD) analysis of RNA duplexes suggest that 8-oxoG modification has little to no effect on the geometry of the A helix adopted by the RNA (61). Similarly, X-ray and NMR analysis of DNA duplexes harboring 8-oxodG•dA or 8-oxodG•dC revealed little to no distortion of the helical structure of the molecule (62–64). However, thermal stability analysis of short modified RNAs shows that the lesion decreases the melting temperature of the 8-oxoG•C duplex by as much as 10°C relative to G•C suggesting that there are energetic penalties associated with this base pair (65). The 8-oxoG•A base pair, in contrast, is significantly stabilized relative to the G•A base pair. Even with this increased stability, the Tm of duplexes containing the 8-oxoG•A base pairs is on average 5°C lower than that of the canonical G•C base pair. These observations suggest that even though the geometry of the 8-oxoG•A base pair is not likely to change the overall structure of the codon–anticodon helix, the energetics of the interaction between the mRNA and tRNA is not as favorable as would be expected for a cognate one.

In comparison to the ribosome, DNA polymerases display varying efficiencies for incorporating dCMP or dAMP opposite to 8-oxodG dependent on the type of the polymerase. For example, replicative polymerases incorporate dCMP across 8-oxodG with frequencies ranging from 1:14 to 90:1 relative to dAMP incorporation (47). In addition, these polymerases are more efficient at extending beyond the lesion when 8-oxodG is base paired with dA relative to dC, suggesting that the polymerases tolerate the mispair presumably due to its similarity to Watson–Crick base pairs. Indeed, structural analysis of DNA polymerases bound with modified primer-template complexes rationalized some of these observed effects on the accuracy of DNA replication as well as the variation in the efficiencies of misincorporation rates (66–68). These studies revealed that both base pairs are accommodated in the active site of the polymerases, but their conformations as well as their interactions with the side chains of the proteins are dependent on the identity of the protein. For instance, in the case of T7 DNA polymerase, the 8-oxodG•A base pair adopts a geometry nearly identical to that of a Watson–Crick base pair, rationalizing the ability of the mispair to escape the proofreading function of the enzyme (69). In contrast to replicative polymerases, translesion enzymes, like those used to replicate over thymine dimers, tend to be relatively more accurate (70). At a structural level, this can be explained by the slightly larger active site employed by these enzymes to allow access for large adduct, which in turn allows for the formation of the 8-oxodG•C base pair. Although we lack equivalent structural data of the ribosome bound to 8-oxoG-containing mRNA, our data suggests that either base pair can form under normal conditions with a preference for 8-oxoG to base pair with A. However, the geometry of the base pair is slightly distorted such that it fails to trigger the required conformational changes, even in the presence of miscoding antibiotics. Given how fast codon recognition occurs, we hypothesize that the rotation of 8-oxoG from the *anti* to *syn* conformation is so rapid that the cognate tRNA has insufficient time to base pair with the mRNA. In the presence of the antibiotic, the cognate tRNA is stabilized long enough for the adduct to adopt the canonical *anti* conformation, activating EF-Tu, and in doing so, suppressing the effect of the modification on tRNA selection.

The ability of ribosomes to bypass oxidative lesions, such as 8-oxoG, may serve as an advantage under oxidative stress conditions. Previous data from our group showed that the presence of 8-oxoG can cause ribosomal stalling and activation of No-Go Decay pathways (45). This stalling generates incomplete peptides that are recognized as such and degraded through proteolysis. In the presence of error-prone ribosomes, we observe increased decoding of the 8-oxoG adduct as either a G or U. Previous work has shown that the ability of error-prone ribosomes to generate mistranslated proteins rather than stall may serve as a signal for the activation of stress response pathways *in vivo* (71). Indeed, *E. coli* that expressed error-prone ribosomes were better able to survive hydrogen peroxide treatment than those expressing wild-type ribosomes. Interestingly, natural *E. coli* vary over 10-fold in their mistranslation rates, suggesting that miscoding is either tolerated or selected for in certain environments (72). The tendency towards error-prone translation in the presence of oxidative damage, such as 8-oxoG, may serve as an important adaptive mechanism through which cells tolerate high-stress environments.

## Supplementary Material

gkz701_Supplemental_File
