# Supplementary material for: Insights into the base-pairing preferences of 8-oxoguanosine on the ribosome
Source: Nucleic Acids Res. 2019 Aug 10;47(18):9857–70. doi: 10.1093/nar/gkz701 (PMC6765139; doi:10.1093/nar/gkz701)
Supplement: gkz701_Supplemental_File [file gkz701_supplemental_file.pdf]

Supplementary Information

Insights into the base-pairing preferences of 8-oxoguanosine on the ribosome

Erica N. Thomas, Carrie L. Simms, Hannah E. Keedy, and Hani S. Zaher*

Department of Biology
Washington University in St. Louis
Campus Box 1137, One Brookings Drive
St. Louis, MO, USA 63130
Phone: (314) 935-7662
Fax: (314) 935-4432

*Correspondence: hzaher@wustl.edu

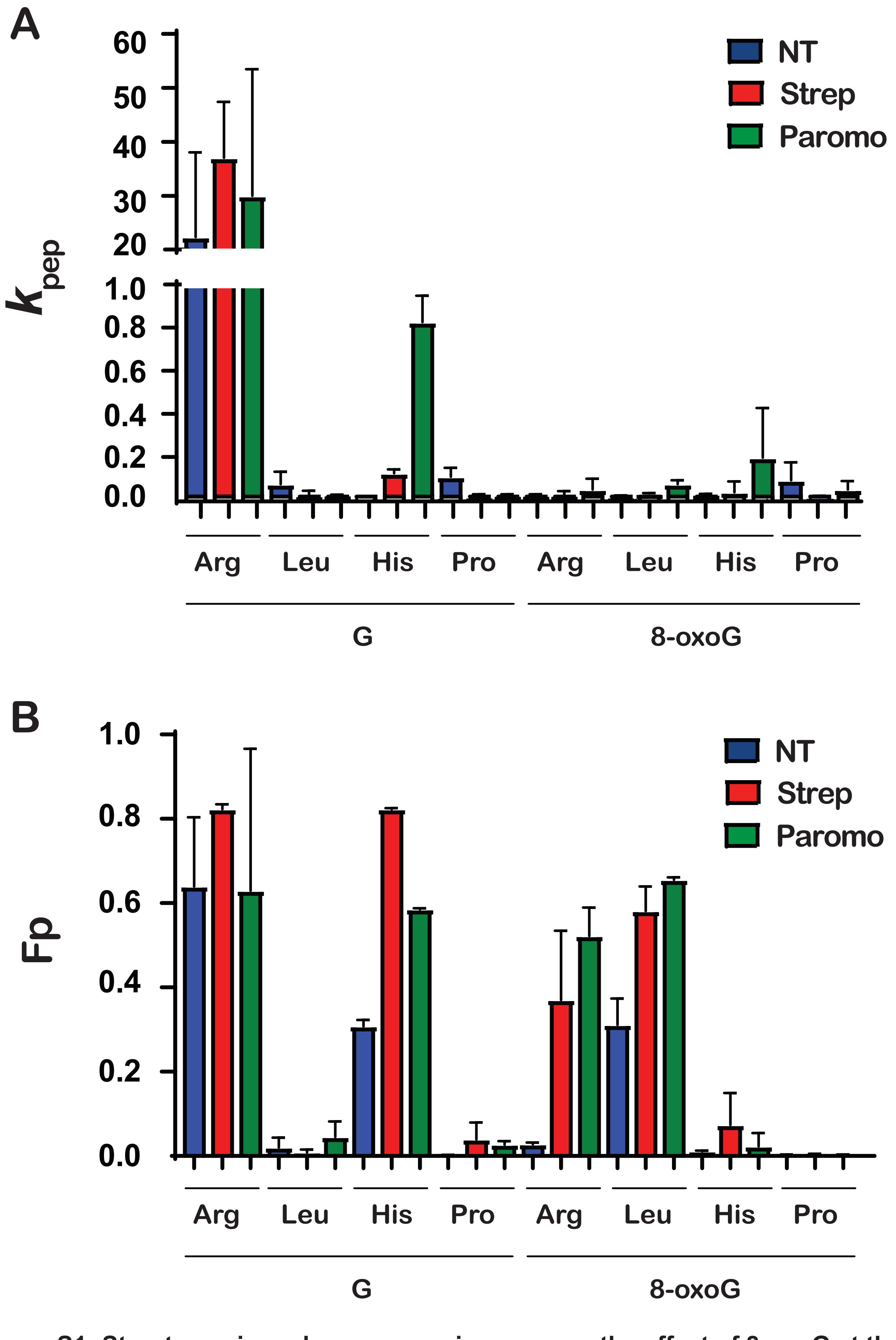

Figure S1: Streptomycin and paromomycin suppress the effect of 8-oxoG at the second position of the codon.

A) Bar graph showing the observed rates of peptide-bond formation (k_{pep}) determined for complexes displaying the CGC (G) or $C^{8oxo}GC$ (8-oxoG) with the indicated ternary complexes.
B) Bar graph showing the end-point values obtained for reactions shown in A. Plotted is the mean of duplicate reactions with the error bars representing the standard deviation around the mean.

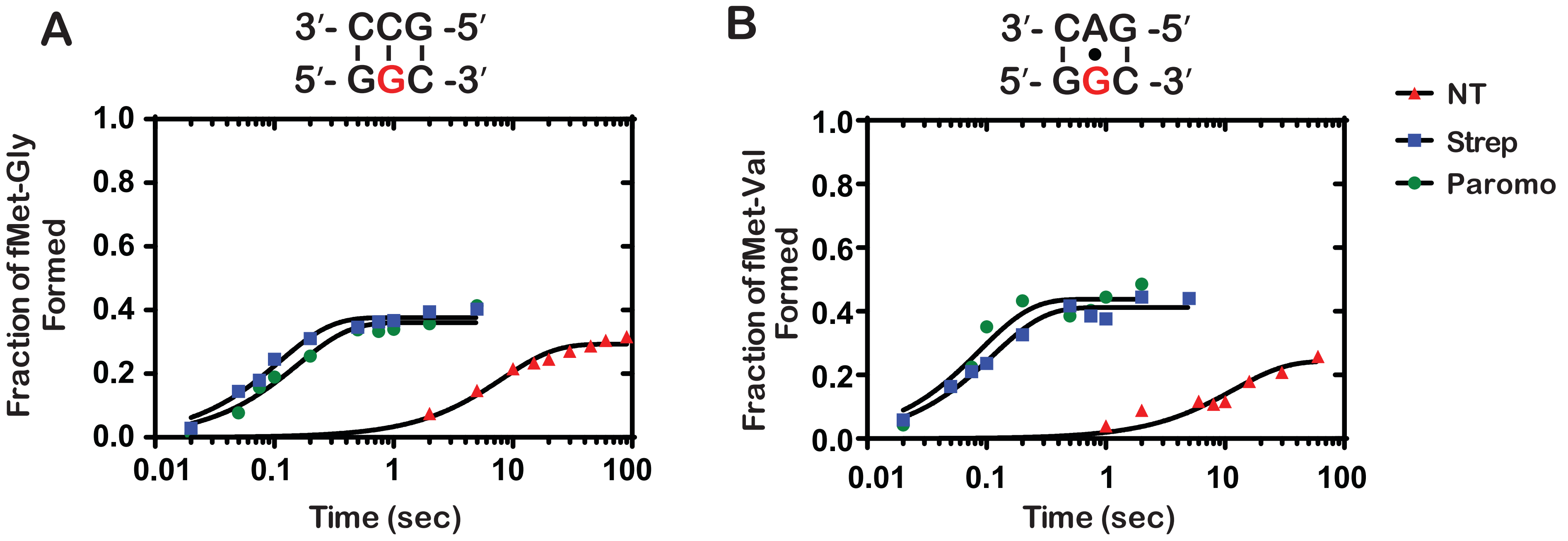

Figure S2: Streptomycin and paromomycin suppress the effects of 8-oxoG on k_{pep} for a complex displaying the $G^{8oxo}GC$ codon in the A site

A-B) Time courses of peptide-bond formation between the indicated initiation and ternary complexes either in the absence of antibiotics or in the presence of paromomycin or streptomycin.

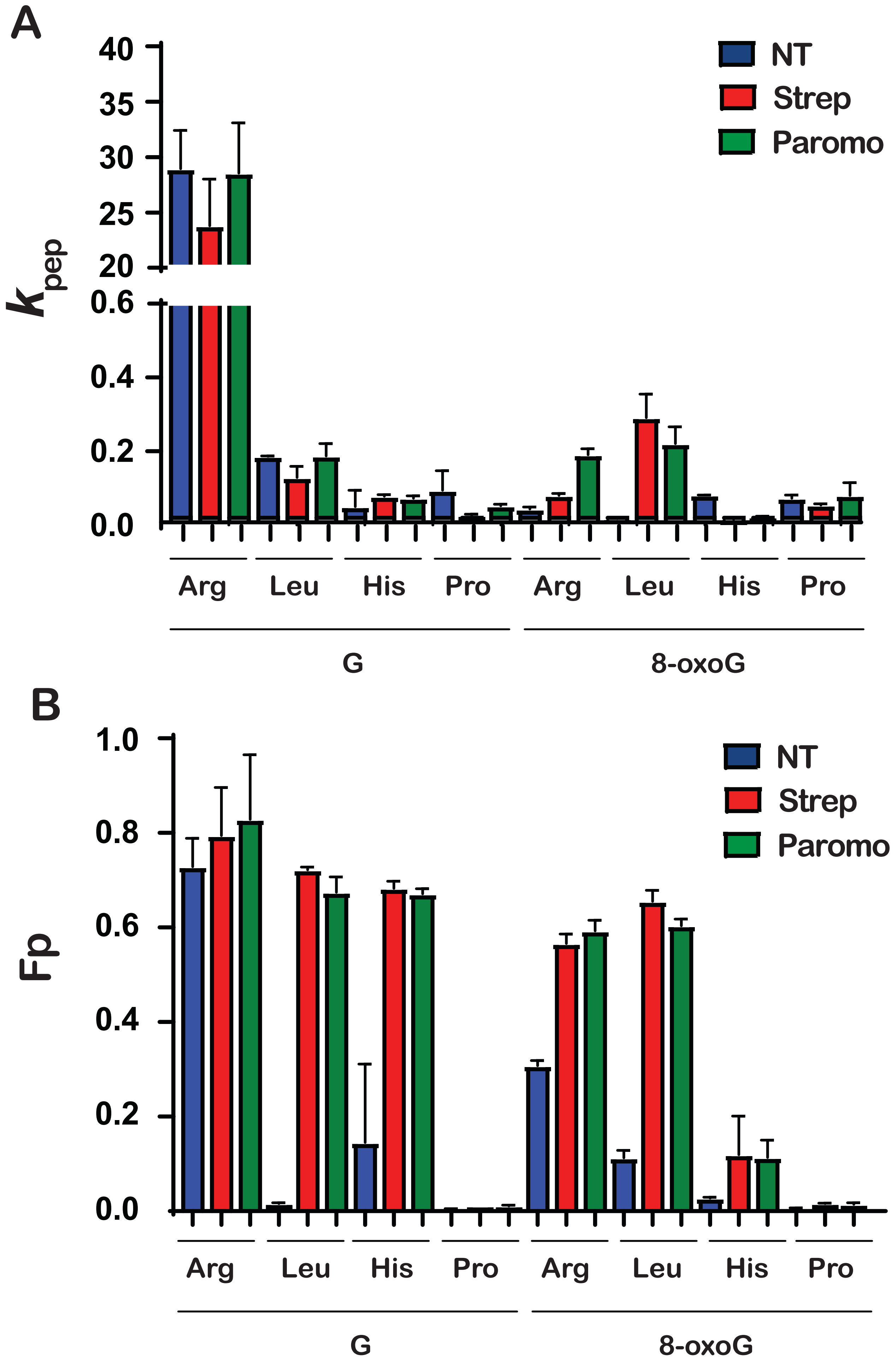

Figure S3: Streptomycin and paromomycin suppress the effect of 8-oxoG at the first position of the codon.

A) Bar graph showing the observed rates of peptide-bond formation (k_{pep}) determined for complexes displaying the GUU (G) or ^{8oxo}GUU (8-oxoG) with the indicated ternary complexes.
B) Bar graph showing the end-point values obtained for reactions shown in A. Plotted is the mean of duplicate reactions with the error bars representing the standard deviation around the mean.
